# Can the Adjustment and Renovation Policies of Old Industrial Cities Reduce Urban Carbon Emissions?—Empirical Analysis Based on Quasi-Natural Experiments

**DOI:** 10.3390/ijerph19116453

**Published:** 2022-05-26

**Authors:** Rongbo Zhang, Changbiao Zhong

**Affiliations:** School of Economics, Shanghai University, Shanghai 200444, China

**Keywords:** old industrial city, carbon emission, green and low carbon path, mechanism path, policy evaluation

## Abstract

Based on a literature review and theoretical mechanism, this paper takes the implementation point of the adjustment and transformation policy for old industrial cities as the breakthrough point, and uses a regression model to explore the impact of the adjustment and transformation policy of these old industrial cities on urban carbon emissions. This paper also robustly tests the effective mechanisms and environmental hypotheses. Overall, the implementation of the adjustment and renovation policy has significantly reduced the carbon emissions of old industrial cities by about 0.068 units. Compared with the control group cities, the pilot cities reduced carbon emissions by an average of about 310,000 tons after the implementation of the policy. Based on a summary of the excellent Chinese case experience and an empirical analysis, it can be concluded that improvements in the green innovation capacity of old industrial cities, the agglomeration of high-end service industries, and the strengthening of ecological restoration are important mechanisms that lead to reduced carbon emissions. There is no subsequent exacerbation of the carbon intensity of neighboring cities, and there is insufficient evidence to prove pollution via neighboring transfers and use of the beggar-thy-neighbor policy. The extended analysis shows that the “inverted U-shaped” CO_2_ Kuznets environmental curve hypothesis is significantly present in the sample of old industrial cities, but most cities do not cross the threshold. In 2013, about 60% of the urban sample economic growth and carbon emissions showed signs of tapping into potentials and increasing efficiency (absolute decoupling) and intensive expansion (relative decoupling). In old industrial cities, the proportion of relative decoupling shows a fluctuating upward trend. In the future, the government should accurately select its own development orientation and actively seek the “best balance” between economic growth and a green and low-carbon path.

## 1. Introduction

General Secretary Xi Jinping has repeatedly emphasized that China’s carbon dioxide emissions will peak before 2030, and the country will strive to achieve carbon neutrality before 2060. Activities related to industrial production and energy consumption are the main sources of carbon dioxide production. Vigorous promotion of carbon emission reduction in the industrial and energy fields is key to the successful implementation of new environmentally friendly development concepts, and to accelerate the construction of a modern industrial system, which in turn will help achieve carbon peaking, and then carbon neutrality. The old industrial cities have made historical and significant contributions to the formation and improvement of an independent and complete industrial system and national economic system in China, and have been indispensable in the post opening-up reform era, and during the current wave of modernization.

The old industrial city, as a basic unit, still faces many difficulties and challenges with the existing institutional mechanisms, policy systems, and governance methods. The past problems remain prominent and cause difficulties for the old industries to adapt to the requirements of the industrial green and low-carbon transformation. Through an in-depth study of the adjustment and transformation of old industrial cities, in the face of today’s good conditions, this paper aims to help accelerate the reform of high-quality economic development and promotion of green and sustainable development of the industrial economy. Promoting regional coordination and complementarity in order to reduce carbon emissions and pollution, are important to the success of building a beautiful, more affluent, healthier society. The policy of adjustment and reconstruction of old industrial bases across the country can be considered a starting point to achieving the environmentally friendly targets. This paper can provide operational, replicable, and scalable experience and practices for the low-carbon development of other industrial cities, and explore new ways for the green and low-carbon transformation of industrial cities across the country.

## 2. Literature Review

Old industrial cities were once the engines that drove rapid economic development in Europe and the USA. “De-industrialization” in the developed countries led to a severe recession. Similarly, due to market system changes, or the over-specialization of industries, old industrial cities will become prominent in developing countries, which will cause serious ecological and environmental problems [1]. How to promote carbon and pollution reduction, especially the green transformation of old industrial cities, with the goal of creating a sustainable “two-oriented society”, is a globally challenging task.

Due to the characteristics of the industrial development cycle, it is harder to achieve green transformation in old industrial cities [2]. They may suffer from resource curse disadvantages that limit their ability to adapt to economic changes, and can cause serious air pollution problems [3]. In terms of the transformation path, old industrial cities have stronger needs than other regions. Trade unions, enterprises, and political figures with greater influence can promote regional green and low-carbon transformation and give play to the comparative advantages of emerging industries [4]. Steel production and heavy manufacturing dominated many of the cities in Pennsylvania. Therefore, the Pennsylvania Steel Authority was founded to address the city’s economic development needs. Strategies employed by the Authority included innovative regional connectivity mechanisms and issuance of early warnings to factories about closures and layoffs [5]. The prosperity of the old Austrian industrial area was based on steel and metal products, but it gradually declined due to a high degree of specialization and weak adjustment capacity; the area recovered through the optimization of regional innovation networks and indirect policy forms [6]. Due to the “industrial lock-in effect”, the competitiveness of traditional Czech industrial zones has declined. Through a low-carbon economy and industrial transformation, the Czech government actively develops modern industries (ICT industry and software industry), which increase the value of the green economy, and stimulate production and employment in low-carbon industries [7]. The French industrial sector promoted sustainable development by introducing a regional cluster plan to reverse its declining industries [8]. The revitalization of the traditional German shipbuilding industry has benefited from policy support and guidance on the development of emerging industries, and multi-level governance from the regional development environment [9]. The old industrial city of Lowell in the USA has accelerated the creative reuse of industrial heritage, and has actively improved the ecological environment of the city to promote the development of new tourism [10]. The key to the transformation of economic development in old industrial cities lies in the transformation of institutional paradigms. The dynamic characteristics of institutions have broad application prospects because of their unique utility.

The process of urbanization and industrialization in China differs from that of Western countries. The analysis of evolution elasticity theory shows that the economic growth rate and industrial structure of old industrial cities can be differentiated [11]. Based on the life cycle theory, and taking the old industrial cities as the research object, the rationalization level of the industrial structure is not found to be high, the economic benefits of most enterprises are marginal, the land is largely idle, the industrial products are at the middle and low end of the value chain, and the innovation capacity is insufficient. The advantages of traditional industries such as iron and steel are weakened, the clean and low-carbon service industry is underdeveloped, the adjustment of industrial structure is slow, and the degree of integration between the service industry and the manufacturing industry is insufficient [12]. The transformation of old industrial cities often carries a serious loss for the young and high-quality labor force, and the burden of an aging labor force becomes more serious. This imbalance in industrial structure leads to structural unemployment, and social security becomes overwhelmed [13]. Shenyang City in Liaoning Province is a heavy industrial city, and the relationship between per capita GDP and carbon emissions is still coupled; the expected decoupling has not yet occurred [14]. As a typical industrial city, China’s Tianjin has more capital investment in industrial infrastructure than real estate, resulting in the largest carbon footprint. Since the early 2000s, Tianjin’s consumption-based carbon emissions due to capital formation have grown rapidly, reaching 72.9%, far exceeding Beijing and Shanghai in China [15]. The single-type old industrial base demonstration area in China, the effect of industrial structure transformation and upgrading, is not good, and the endogenous driving force for transformation and development is also weak [16]. Therefore, the transformation of industrial cities in the future should comprehensively deepen reforms, emancipate ideas, and create a stable policy environment. Old industrial cities should break down the institutional barriers that hinder its revitalization and focus on new economic services and high-end industrial modernization. The old industrial cities should accelerate towards a high-end low-carbon service-oriented economy and increase the added value of industrial green products [17].

The existing literature mainly comprises a factual elaboration and theoretical summary of the old industrial cities, and discusses their implementation path and future direction from the perspective of case analyses. However, less empirical attention has been given to the net impact of the adjustment and comprehensive transformation of old industrial cities on regional green and low-carbon development.

Given this, the possible contributions of this paper are:

(1) Evaluate the low-carbon effects and spatial spillover effects with the adjustment and renovation policies of old industrial cities.
(2) Effectively identify the transmission mechanism by which the adjustment and transformation of old industrial cities can help reduce carbon and pollution.
(3) Test whether a curve is significantly established in the old industrial city sample based on the Kuznets inverted U-curve theory of carbon dioxide.
(4) To analyze the degree of decoupling of old industrial cities.

**Figure 1:**
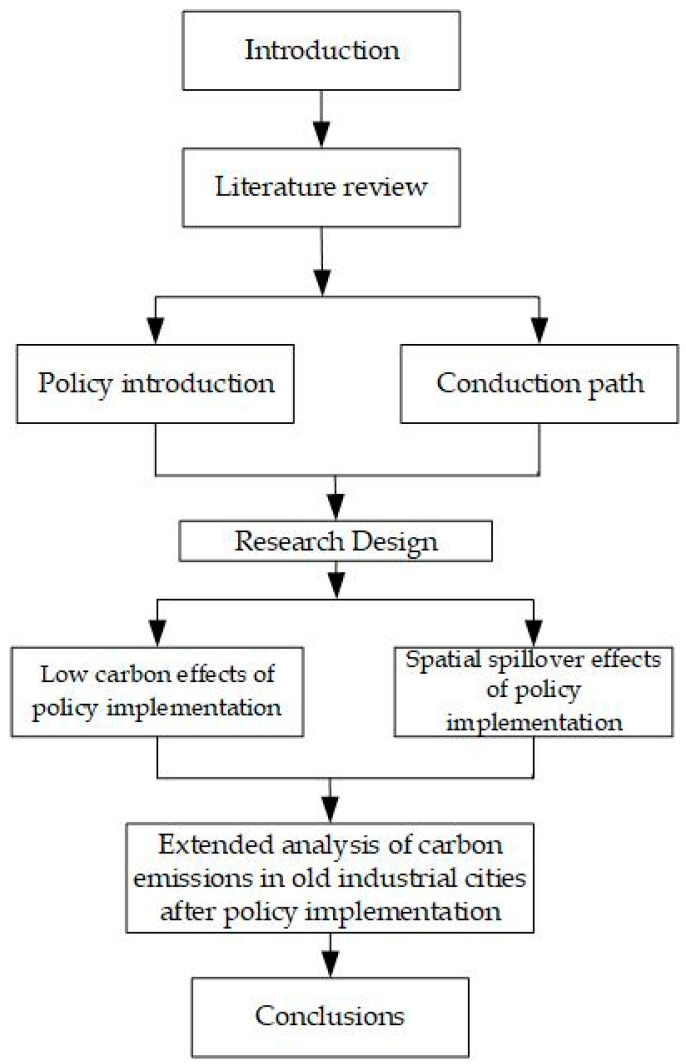
Flow chart of research framework.

We give a flow chart of the research framework of the article. The flow chart of the research framework is shown in Figure 1.

## 3. Policy Introduction and Theoretical Mechanism

### 3.1. Policy Introduction

Achieving carbon peaking and carbon neutrality are necessary to solve the outstanding problems of resource and environmental constraints, and to ensure sustainable development, in turn nurturing China’s lucid waters and lush mountains. China’s “Notice of the State Council on Printing and Distributing the Action Plan for Carbon Peaking Before 2030” identified the need to establish an economic system of green, low-carbon, and circular development by 2025. The Notice also specifies that carbon dioxide emissions per unit of GDP will drop by 18% compared to 2020, and that substantial progress will have been made in the comprehensive green transformation of economic and social development, with carbon dioxide emissions reaching a peak and achieving stable and moderate declines by 2030. The government encourage key fields such as energy, industry, transportation, and construction to formulate special plans for peaking, and promote key industries such as steel, building materials, nonferrous metals, chemicals, petrochemicals, electric power, and coal to put forward clear peaking goals and formulate peaking action plans. By 2060, the goal of carbon neutrality will be successfully achieved, and a green, low-carbon, and cyclical economic system and a clean, low-carbon, safe, and efficient energy system will be fully established, creating a new realm of harmonious coexistence between man and nature.

Old industrial cities face dual pressures in the process of carbon reduction due to their own unique realities. The proportion of traditional industries is still relatively high, and it is increasingly difficult to tap the potential of energy conservation. The coal-biased energy structure, inefficient energy utilization, and low utilization of clean energy remain apparent. The lack of advanced technology reserves, the shortcomings of energy-saving and efficiency-enhancing technological innovation, the tight time window for carbon peaking and carbon neutralization, and the arduous task of realizing the green and low-carbon transformation of old industrial cities, are the unwanted features that still dominate old industrial cities.

In order to promote energy conservation, emission reduction, and the sustainable development of old industrial cities, the reform plan for renovating these cities should focus on transforming the mode of economic growth and promoting sustainable development. The government focusing on transforming the mode of economic growth and promoting sustainable development, focusing on the new competitive advantage of re-engineering the industry, taking promoting green and low-carbon development, and enhancing innovation support capabilities as an important focus, promote the comprehensive, coordinated, and sustainable development of old industrial bases, building it into an important new low-carbon industrial base for the country and a key growth pole for the high-quality development of the regional economy.

### 3.2. Conduction Path 

By considering the success rate of the measures taken by 94 old industrial cities after the implementation of the adjustment and renovation policy, their transmission path is proposed. Representative cities in the eastern region (Zibo City, Shandong Province, China), central region (Changzhi City, Shanxi Province, China), and western region (Baiyin City, Gansu Province, China) were then selected for case analysis.

#### 3.2.1. Green Innovation Capability

Industrial green innovation. The continuous innovation of urban intelligent green manufacturing technology can help transport an industry to the high end of the value chain, change the existing unsustainable technology and production system, and improve the level of informatization and automation of the manufacturing industry. The government can deepen the degree of coupling between the innovation chain and the industrial chain; increase the transformation of innovation achievements and the research and development, as well as the application of green and low-carbon cutting-edge technologies; accelerate the upgrading of low-carbon products and technologies; and form new manufacturing processes and equipment that are efficient, energy-saving, environmentally friendly, and recyclable, building a green zero-carbon manufacturing system, thereby greatly reducing carbon emission intensity. Industrial technological innovation can broaden the depth and breadth of the development and utilization of renewable clean energy and promote a revolution in energy consumption. Industrial green innovation also reduces excessive dependence on fossil fuels, optimizes energy industry structure and energy use ways, and improves the efficiency of the optimal allocation of energy industrial cities. The government accelerate the conversion of old and new kinetic energy in cities, and continuously overcome key energy-saving technologies in major energy-consuming fields of industry, so as to reduce total carbon emissions from the source [18].

Environment for innovation activities. An environment conducive to innovative scientific research and the construction of innovative service networks plays an increasingly important role in carbon emission reduction. Scientific research includes technological projects in the fields of energy conservation and environmental protection, low-carbon production, and clean energy, and forces and/or motivates enterprises to develop advanced production technology. The people in old industrial cities participate in and popularize the practice of green production and life, actively advocating green, low-carbon, and simple lifestyles in the whole society, and indirectly reduce the scale of carbon emissions [19].

#### 3.2.2. High-End Industry Agglomeration

Efficient division of labor. Emerging high-end industrial clusters can reduce production and trade costs through the specialized division of labor and advantageous resource endowments, in turn effectively promoting business exchanges between enterprises. This will help reduce the volatility of demand for green and low-carbon products, deepen the division of labor in the industrial chain, and promote the extension of the industrial chain towards high added value [20].

Element sharing. The agglomeration of high-end industries is conducive to promoting the effective allocation of production factor resources and recycling urban infrastructure. Element sharing helps to reduce the transportation distance, expenditure cost, and time search cost in old industrial cities. Element sharing can improve the government’s treatment and utilization efficiency of pollutants, reduce the decentralized consumption of energy, improve the utilization efficiency of energy and elements, and thus promote the rapid reduction of urban carbon emissions.

Spillover effect. The complementary effect of the modern low-carbon industry and its related industries will produce increasing returns to scale, resulting in the multiple effects of urban collaborative innovation, cutting-edge ideas, and spillover enhancement effects. The spillover effect will help to enhance the rooting and cohesion of the low-carbon industry, and increase the communication, interaction, and cooperation between enterprises and other related industries. The diffusion of urban clean technology will accelerate, and upstream and downstream enterprises will be promoted through exchanges and interaction along the industrial chain. In addition, other companies will benefit from the agglomeration of high-end low-carbon industries [21].

#### 3.2.3. Ecological Governance

Firstly, ecosystem protection and restoration can significantly increase the coverage of natural vegetation, prevent soil erosion and dust storms, increase soil fertility, expand the total amount of forest and grass resources, maintain biodiversity, and effectively improve regional air quality. Ecological governance can change local albedo, turbulent energy, and mitigate natural disaster losses. Ecological governance can promote the enhancement of the carbon sequestration and carbon absorption capacity of forests and wetlands, and effectively reduce the total carbon emissions of cities. Afforestation programs that promote increased terrestrial carbon storage are an important means of helping to gradually reduce atmospheric CO_2_ emissions [22].

The research on forest carbon sinks of Peking University also shows that for every cubic meter of forest growth, it absorbs about 1.8 tons of carbon dioxide on average, especially in the peak season of tree growth, the forest can absorb 1.6 kg of carbon dioxide per square meter per day. The United Nations Intergovernmental Panel on Climate Change estimates that the global forest carbon storage will exceed 11,000 tons. In order to achieve carbon neutrality, cities require conservation as well as sustainable management to increase carbon sink. Therefore, old industrial cities must formulate policies against deforestation and deforestation, promote scientific research on carbon sinks, actively build forest parks, and promote afforestation activities [23]. Research on the forest carbon sinks of Peking University also shows that for every cubic meter of forest growth, an average of 1.8 tons of carbon dioxide are absorbed; the forest can absorb 1.6 kg of carbon dioxide per square meter per day during the peak season of tree growth.

Secondly, the expansion of the regional green area can optimize the urban ecological space pattern, enrich the urban connotation and functional quality, and continue to attract new workers. Profiting from their industrial heritage resources, cities can provide a high-quality creative environment and atmosphere through exhibitions and projects, creating green and low-carbon development ecological places, tourist attractions, and idyllic scenery complexes. Ecosystem protection will help cities fundamentally transform their original industrial structure of high energy consumption, high pollution, and high emission industries, realizing the integrated and diversified development of primary, secondary, and tertiary industries, continuously improving the “green content” of emerging industries, and accelerating the realization of low carbonization [24].

## 4. Research Design

### 4.1. Methods and Variables

DID model. The difference-in-differences method is an important scientific research tool for evaluating the effect of a project or policy implementation. This method can not only exclude unobservable individual heterogeneity among samples, but also account for the influence of unknown factors that change over time. The difference-in-differences method ensures unbiased estimation of policy effects and effectively controls the effect of the interaction between the explained variable and the explanatory variable. Based on the pilot list of old industrial cities, the benchmark regression model is constructed as follows:

$$Y_{it}=\zeta +\gamma_{1}DID_{it}+\gamma_{2}Oinvest_{it}+\gamma_{3}Pergdp_{it}+\gamma_{4}Oeduc_{it}+\gamma_{5}Upais_{it}+\gamma_{6}Dmarket_{it}+\gamma_{7}Teleinst_{it}+\vartheta_{i}+\psi_{t}+\mu_{it}$$

where by i is the old industrial city, and t represents the year; Y represents the dependent variable; and the key explanatory variable $\gamma_{1}$ represents the net impact of the adjustment and renovation policy of the old industrial city on urban carbon emissions. Models control for time and city fixed effects, add random distractors, and estimate using city-level cluster robust standard errors. The changes-in-changes (CIC) model. The CIC model by Athey [25] is used as a reference for testing, and may either be considered an extension of the quantile treatment effect model over multiple periods or a formal generalization of the standard two-difference model, as it relaxes many of the assumptions of the standard model.

Panel quantile regression model. The basic idea of this model is to minimize the distance between the dependent variable and the fitted value, which can effectively eliminate the assumption of a normal distribution for the unobserved residuals based on the minimum residual squared and panel model, and better measure the independent variables in the variation trends and distribution shapes at multiple quantiles, which reflect the rich information of all samples.

The parameter estimation method of the panel quantile regression model is as follows:

$$\overset{\wedge }{[\beta }(\tau_{k},\lambda ),{\left\{\alpha_{i}(\lambda )\right\}}_{i=1}^{n}]=arcmin\sum_{k=1}^{K}\sum_{l=1}^{T}\sum_{i=1}^{N}\omega_{k}\rho_{\tau k}(y_{it}-\alpha_{i}-x_{it}^{T}\beta_{\tau k})+\lambda \sum_{i=1}^{N}\left|\alpha_{i}\right|$$

where $\omega_{k}$ is the weight of the quantile $\tau_{k}$ and $\tau_{k}$ represents the contribution of the quantile k to the fixed effects. $\rho_{\tau k}(u)=u[\tau_{k}-I(u<0)]$ is the loss function and I(u<0) is the indicative function. λ is the penalty factor; when λ<0, the penalty term disappears, and it is a fixed effect model; when λ tends to infinity, the fixed effect disappears, which is a mixed regression model. The model considers the effect of subjectivity on weight assignment. The model should define a weight $\omega_{k}=1/k$; that is, assign the same weight to all indicators, thereby reducing the impact of subjectivity.

Explained variable: total urban carbon emissions (*Lnco*2). Referring to the calculation method of Wu Jianxin [26], urban carbon emissions include not only carbon emissions from direct energy consumption, such as coal gas, natural gas, and liquefied petroleum gas, but also indirect carbon emissions from electricity, heat, and urban transportation. Sum the two and take the logarithm to get the city’s total carbon emissions. In addition, per capita carbon emissions *Perco*2 (the ratio of the total urban carbon emissions to the urban population) and land average carbon emissions *Aerco*2 (the ratio of the total urban carbon emissions to the land area of urban administrative units) are both estimated as dependent variables.

Direct energy carbon emissions are obtained by multiplying the total urban natural gas and LPG supply by the relevant conversion factors provided by IPCC2006. The CO_2_ emission coefficient of natural gas is 2.1622 kg-CO_2_/m^3^. The CO_2_ emission coefficient of LPG is 3.1013 kg-CO_2_/kg. The CO_2_ emission coefficient of raw coal is 1.9003 kg-CO_2_/kg. There are six regional power grids in China: North China, Northeast China, East China, Central China, Northwest China, and South China. The carbon emissions generated by urban electric energy consumption are calculated by multiplying the annual emission factors of the regional grid baselines and the urban electric energy consumption (the electricity consumption of the whole society). The carbon emission of urban transportation is based on the methods of Zhang Yan [27] and Zhao Min [28]. It is assumed that the speed of urban public transportation is 16 km/h, the daily driving time is about 12 h, and the fuel consumption per 100 km is 32 L/100 km. The annual driving mileage of urban taxis is 12,000 km/year, and the fuel consumption per 100 km is 10 L/100 km. The gasoline emission factor provided by the IPCC guidelines is 6.93 × 10^4^ kg/TJ (as CO_2_). The net calorific value of gasoline is 44.3 TJ/Gg, which is 44.3 × 10^−6^ TJ/kg. The diesel emission factor is 7.41 × 10^4^ kg/TJ (as CO_2_). The net calorific value of diesel is 43 TJ/Gg, which is 43 × 10^−6^ TJ/kg. The emission coefficient of raw coal is 9.46 × 10^4^ kg/TJ (calculated as CO_2_), and the net calorific value is 25.8 TJ/Gg, which is 25.8 × 10^−6^ TJ/kg. The urban public transportation uses diesel as the main fuel, and the density of diesel is 0.835 kg/L. Taxis use gasoline as fuel, and the density of gasoline is 0.725 kg/L. The urban thermal energy data were not taken into account due to the large area of missing values in the total urban heating supply and heating area.

Core explanatory variables. $DID_{it}=treat_{i}*post_{t}$ is the core explanatory variable. If the city is a pilot city involved in the old industrial adjustment and renovation policy, then $treat_{i}=1$. When t<2013, $post_{t}=0$.

Other explanatory variables: Economic development (*Pergdp*), as measured by the ratio of urban real GDP to total urban population. GDP is an important factor affecting CO_2_ emissions in old industrial cities. With the continuous increase in the total economic volume and output scale, the new emission of pollutants has become prominent, which has brought great pressure on the emission reduction of old industrial cities. Investment level (*Oinvest*), as expressed by the ratio of the whole society’s fixed asset investment and real GDP. The impact of investment levels on the green and low-carbon development of old industrial cities is uncertain. On the one hand, old industrial cities have increased investment to attract “high energy consumption, high pollution, high emissions, low added value” enterprises to enter, and constantly reduce the quality of the city’s green development. On the other hand, the increasing investment in environmental protection has provided effective financial support for eliminating the pollution stock and reducing the pollution increment. The government’s efficient environmental protection investment can promote the reduction of the county’s pollution emission intensity. Education scale (*Oeduc*), which is represented by the ratio of the number of students in regular high schools in the city to the urban population. The scale of education can significantly reduce the scale of carbon emissions in old industrial cities and increase the carbon sequestration of regional vegetation. Education has a reinforcing effect on public environmental protection behavior in old industrial cities. Education carries the universal function of social values and can increase knowledge about ecological and environmental protection. Industrial structure (*Upais*), as represented by the advanced index of urban industrial structure, that is, $Upais_{it}=W_{1it}\times 1+W_{2it}\times 2+W_{3it}\times 3$, W represents the ratio of the first, second, and third industries in the city i to the total GDP in t period. The advanced transformation of the industrial structure can speed up the research and promotion of industrial low-carbon technologies and explore new models of low-carbon industrial development. The progress of industrial structure can enhance industrial technological innovation in old industrial cities, improve the manufacturing of urban industrial green products, develop a low-carbon green economy, and reduce regional carbon emissions. Marketization level (*Dmarket*), as represented by the ratio of the number of private and individual employees in the city to the total number of employees in the city. The improvement of market demand is conducive to the public consciously transforming green concepts into actual consumption actions and building a social action system for low-carbon consumption. To a certain extent, it is conducive to industrial structure adjustment and independent innovation, and guides enterprises to actively adapt to the requirements of green and low-carbon development. Communication facilities (*Teleinst*), as represented by urban fixed telephone users and mobile users. The rapid increase in the coverage of communication facilities can prompt the public to spontaneously monitor the environmental pollution around living areas, exert high pressure on local governments, and increase the quality and scope of government environmental pollution information disclosure. The multicollinearity test found that the largest covariate VIF was 2.56, 1/VIF was 0.91, and the mean VIF was 1.83. There was no serious multicollinearity problem.

### 4.2. Samples and Data

The article uses panel data of mainland China cities from 2006 to 2019. A list of the policies for adjustment and renovation of old industrial cities can be obtained from the official website of the National Development and Reform Commission. Other data were obtained from the “China Urban Statistical Yearbook”, “China Urban Construction Statistical Yearbook”, “China Regional Economic Statistical Yearbook”, “China Electric Power Yearbook”, “China Energy Statistical Yearbook”, and provincial (municipal and autonomous region) statistical yearbooks over the years. If there was a problem of missing data, the CSMAR database and EPS database were used as alternatives. The descriptive statistics of the variables are shown in Table 1.

### 4.3. Time Trend and Comparative Analysis

**Figure 2:**
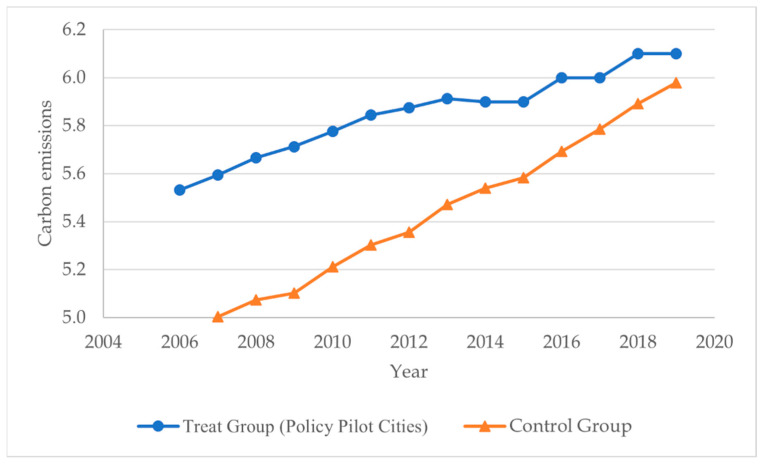
2007–2019 carbon emission trend chart of Treatment Group and Control Group cities.

By selecting the time series data from 2006 to 2019, the annual change trend in urban carbon emissions in the treatment group and the control group was analyzed before and after the implementation of the policy. According to Figure 2, the following conclusions can be drawn. From the perspective of the time trend, compared with the control group cities, the urban carbon emissions in the treatment group cities dropped significantly at an average rate of 0.18 units after the implementation of the old industrial city adjustment and renovation policy. From the comparative analysis of the cities in the treatment group and the control group, before 2013 (the year the policy was implemented), the carbon emissions of the cities in the treatment group and the control group basically showed a parallel trend. After the implementation of the policy, the carbon emissions of the cities in the treatment group showed a significant and rapid downward trend. Through the analysis, it is preliminarily shown that the low-carbon effect of the implementation of the adjustment and reconstruction policy of the old industrial cities on the pilot cities is real and effective.

## 5. Estimated Empirical Results

### 5.1. Regression Analysis

This paper considers the impact of the adjustment and renovation policies of old industrial cities on regional carbon emissions. The benchmark regression estimation results in Table 2 show that the estimated coefficient of Model (1) is negative, and has passed the 5% statistical significance test, which preliminarily demonstrates that the adjustment and reconstruction policy will suppress the amount of carbon emissions in the old industrial cities, and thus improve ecological and environmental protection, and the efficiency and benefits of regional ecological development; carbon dioxide pollution levels will also be better controlled. Irrespective of whether or not the control variable is added, and whether or not it is clustered in cities, the negative significance of the core parameters still holds true, and the estimated coefficient is stable at −0.068. It shows that the adjustment and renovation policy of old industrial cities has a significantly negative effect on regional carbon emissions, which causes a positive externality of the ecological environment. Unlike the control group, policy implementation can promote the average reduction of 0.068 units of total urban carbon emissions in the treatment group. From an economic viewpoint, the adjustment and renovation policies of old industrial cities have reduced urban carbon emissions by about 310,700 tons.

Before the implementation of the old industrial city adjustment and renovation policy, the old industrial city exceeded the threshold of the carrying capacity of the social environment through the excessive consumption of energy resources, and substantial investment in related factors of production, which all reduced the stability and service functions of the regional ecosystem. Following policy implementation, the original economic development model of the old industrial city has been gradually broken. The old industrial cities, according to the resource endowment conditions and the main function positioning, anchors the green and low-carbon development plan, leading to deeply adjusting the industrial structure and making every effort to promote industrial energy conservation and emission reduction. The old industrial cities can accelerate industrial innovation and low-carbon construction projects, strengthen the hard constraints of the ecological environment, and promote the elimination of backward and excess production capacity. Old industrial cities actively explore new models of low-carbon industrial development and vigorously develop low-carbon modern service industries. Additionally, old industrial cities are accelerating the continuous reduction of energy consumption per unit of product, strengthening their recycling capacities and comprehensive utilization of resources, and promoting manufacturing and supply of green products. Old industrial cities can strictly abide by the red line of urban ecological protection, increase the carbon sinks in the ecosystem, resolutely curb the blind development of high energy consumption, and optimize the layout of land and space development and protection areas. To sum up, the implementation of the policy can effectively reduce the emission intensity of carbon dioxide pollutants in cities and promote the green and low-carbon development of cities.

According to Table 2, the scale of education and communication facilities significantly reduce the level of carbon emissions in the region, whilst economic development has an inhibitory effect on the green and low-carbon effect of the city.

The implementation of the new development concept has enabled old industrial cities to gradually incorporate ecological civilization education into the national education system, which has strengthened innovation capacity building and talent training, incited green and low-carbon social actions, and popularized basic knowledge of carbon peaking and carbon neutrality. With the implementation of the new development concept, old industrial cities have gradually incorporated ecological civilization education into the national education system, strengthened innovation capacity building and talent training, and led to green and low-carbon social actions. People’s consumption habits, ecological awareness, and market demand are constantly changing, resource conservation is increasing, and the degree of public participation is orderly. The coverage network of regional communication infrastructure can significantly enhance public participation in environmental governance, and enhance the environmental protection demands of informal forces. The advancement of communication facilities has significantly improved people’s ideas and ways of thinking and boosted the rapid flow and efficient dissemination of diverse information. The coverage network of regional communication infrastructure can improve the availability of products and services in the region and help enterprises to make optimal production and sales decisions, thereby improving industrial production efficiency and reducing urban carbon emissions. In addition, the extensive way of economic development in old industrial cities negatively increases the pollution of the ecological environment and significantly increases the total regional carbon dioxide emissions. With the continuous increase in the total economic volume and output scale, the lagging industrial structure and the increasing risk of spatial layout, the synergy of the development of modern low-carbon industries needs to be improved, and the pressure of new pollutant emissions is prominent. With the weakening of environmental protection, the overall coordination of ecological protection has increased, and the comprehensive deepening of the reform has not yet formed a system integration effect.

**Figure 3:**
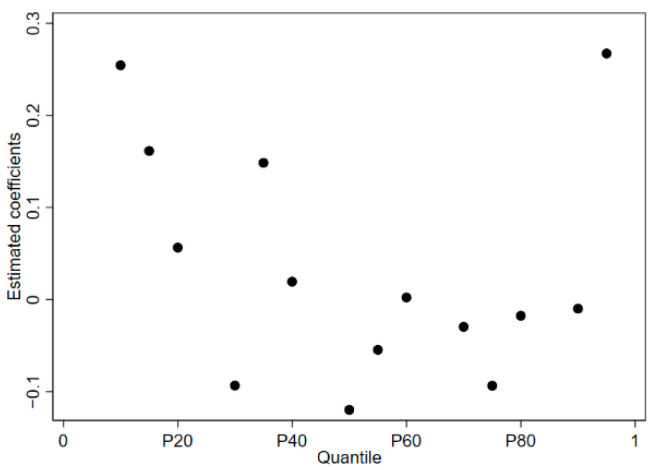
Estimated coefficients of different quantiles.

According to Figure 3, taking the total urban carbon emissions as the explained variable, the key estimated coefficients show a trend of fluctuation first and then decreasing with the increase in quantiles.

Overall, at the lower quantiles, the old industrial cities lack protection from mountains, rivers, forests, fields, and lakes, and the ecological space is instead constantly being eroded and occupied. High development intensity comprises the expansion of urban construction land, increased traffic density, continuous population growth, and intensification of the area of natural vegetation. Per capita consumption of energy-intensive products continues to rise, increasing the city’s carbon emission levels. With the continuous increase in quantiles, the old industrial cities should start from improving the overall competitiveness of the region, clarify their own ecological function positioning, and strengthen the dual control of carbon emission intensity and total amount. The old industrial cities can adhere to the combination of industrial transfer and sustainable development, innovate the way and platform for undertaking industrial transfer, and improve the layout management of low-carbon industrial development and the ecological space within the region. Old industrial cities can optimize the carrying capacity of resources and environment, implement integrated protection and restoration, and consolidate the role of ecosystem carbon sequestration, thereby producing a positive green and low-carbon effect. At the highest quantile, the old industrial cities have heavy work tasks to reduce production capacity, adjust their structure, and change their methods, as there are serious historical problems, and the process of industrial transformation, upgrading, and optimization is relatively slow. The land use, energy consumption, water consumption, factor allocation, and pollutant discharge standards undertaken by the industry are relatively weakened, and it is easy to “turn one eye and close one eye” in order to continue to “give the green light” for pollution-transfer enterprises. Old industrial cities neglecting the quantity and quality of investment promotion will lead to the misallocation of production factor resources and bring about regional ecological and environmental crises.

### 5.2. Robustness Test

**Figure 4:**
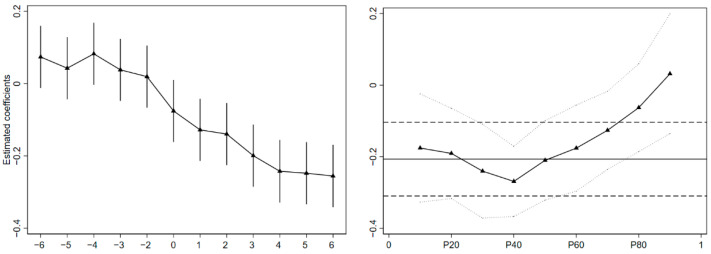
Parallel trend test and CIC test. Note: The picture on the left is the parallel trend test. In the figure, the abscissa “−2” represents the 2nd year before the implementation of the policy, and “2” represents the 2nd year after the implementation of the policy. The ordinate represents the estimated coefficients. The picture on the right is the CIC test. The article uses data from six years before and after policy implementation for analysis. The abscissa represents the quantiles. The vertical axis represents the estimated value of the treatment effect. Solid lines with small triangles represent estimated coefficients at different quantiles. The solid horizontal line is the mean CIC estimate. The horizontal dashed line is its 95% confidence interval.

Following the omitted variable test, the replacement variable test (explanatory variables lagged by one period; explained variable is abbreviated by 1%; Transforming Urban Carbon Emission Explained Variables [29]), the benchmark variable test (a time linear trend (the squared term of the time trend) and an interaction term for urban characteristics (cities in the northern region, cities on the left side of the Hu Huanyong line, top 100 cities in China’s Green Cities Competitiveness Index, resource-based cities, high-speed rail cities, cities with impoverished counties, inter-provincial border cities, sub-provincial city, and capital city) were added to the model), the instrumental variable test (the interaction term between provincial industrial added value (1965–1978) and whether it is a “third-tier construction” city) [30], the parallel trend test, the CIC test, PSM-DID test (DID estimation after Nearest Neighbor Matching and Kernel Matching), the group swap test (swap treatment and control samples, perform DID estimation again), and the policy confusion test (delete sample cities for low-carbon city pilot policies) were ran; the conclusions obtained from the test results are not significantly different from the previous empirical results. It fully shows the reliability of the benchmark estimation results. The empirical estimation results are shown in Table 3 and Figure 4.

### 5.3. Heterogeneity Analysis

As old industrial cities differ with regards to geographical location, administrative level, social endowment, and regional attributes, the impact of policy implementation on urban carbon emissions will also differ. Therefore, in terms of geographical location, the cities were divided up into the eastern region, central region, and western region; in terms of administrative levels, they were grouped by size as large cities, medium-sized cities, and small cities; in terms of convenience, they were divided into cities with high-speed rail and cities without high-speed rail. The estimated results are shown in Table 4.

#### 5.3.1. Impact on Geographic Location

Compared with cities in the eastern and western regions, the adjustment and renovation policies of old industrial cities in the central region have not produced significantly positive regional green and low-carbon effects. Due to the complexity and particularity of the old industrial cities in the central region in terms of geographical location and social environment, it is easy to undertake the transfer of polluting industries and increase the level of carbon emissions. Overcapacity and low-efficiency in central cities still plague the process of industrialization reform. Influenced by interestism, the central region government relaxes environmental control and lowers the regional requirements for environmental quality, thereby ignoring ecological and environmental planning and protection in the region.

The challenge of ecological environmental protection is thus increasing; hence, there is no evidence of positive pollution and carbon reductions. The old industrial cities in the eastern region actively exert their own resource endowment advantages, strengthen industrial environmental protection awareness and low-carbon concepts, and accelerate their industrial innovation capabilities and the training of high-quality talents. Old industrial cities in the eastern region are strengthening the research, promotion, and application of major low-carbon technologies to overcome the commonalities and key core technologies. In addition, the estimated effect coefficient generated by the implementation of the old industrial city policy in the western region is the largest. The old industrial cities in the western region are striving to promote the green, circular, and low-carbon development of the industry, actively exploring the environmental supervision, regulation mechanism, and innovative transformation path of the industrial wasteland, and steadily promoting the formation of a new model of green, low-carbon, and sustainable development. The western region is generally establishing a resource-saving and environmentally friendly development mode to further strengthen the main functions of the different regions.

#### 5.3.2. Impact on Administrative Hierarchy

For large old industrial cities, the implementation of adjustment and renovation policies can have a very significant effect on reducing pollution and carbon in the region. Therefore, large-scale city governments are using “carbon peaking and carbon neutrality” as the guide, and green and low-carbon industrial clusters as the carrier. In taking this approach, large cities will continue to adjust and optimize the regional layout of industrial productivity, guide a rational and orderly transfer of industries, have efficient and clean agglomeration, improve the coordination and supporting system of the industrial chain, create a circular industrial entity, and improve the level of industrial informatization. Large cities are accelerating the research, development, and promotion of industrial low-carbon technologies, exploring and innovating low-carbon industrial development models, enhancing independent innovation capabilities, improving industrial technology innovation mechanisms, promoting in-depth implementation of industrial green manufacturing, and developing an innovative green economy. Large cities are turning “green” in the “background” of industrial production, taking the lead in achieving carbon peaks with a more proactive attitude.

#### 5.3.3. Impact on Convenience

Compared to old industrial cities without high-speed rail, those with high-speed rail have a significantly “negative shadow” impact on their urban carbon emissions, which is conducive to reducing carbon levels and pollution, reducing costs, and increasing efficiency in the region. The reason is that the opening of high-speed rail can improve the accessibility level and location advantages of a city. Transportation times and efficiency increase the potential of agglomeration and economies of scale in the central area, and change the spatial distribution pattern of the urban industrial economy. High-speed rail cities continue to effectively promote the optimization of the industrial division of labor, and the complementation of comparative advantages in the region promote the continuous conversion of new and old kinetic energy, thereby greatly reducing the intensity and total amount of carbon emissions.

## 6. Expansion and Discussion

### 6.1. Discussion on the Mechanism Path

Green innovation quality (*Grinnov*). Patents have become a popular way for researchers to measure green and low-carbon innovation. Therefore, with reference to Zhang [31], it is represented by the number of urban green patents. The empirical results in Table 5 show that the government of the old industrial city is focusing on low-carbon technology and key research and development, establishing and improving the production system of green and low-carbon circular development, accelerating the breakthrough of technological bottlenecks in the industrial industry, and building a market-oriented green technology innovation mechanism. The scientific and technological innovation strategy to promote low-carbon transformation has achieved initial results in samples of old industrial cities, which lays a solid technical foundation for achieving carbon emission reduction goals in the future [32].

Agglomeration of modern industries (*Hinsera*), drawing on the practice of Xuan [33] to build the index. “High-end industries” are industries with high-tech content, high industry-leading capabilities based on high professional human capital investment, and high tacit knowledge spillover characteristics. The test results in Table 5 show that high-end industrial agglomeration helps to efficiently promote close cooperation between enterprises, integrate knowledge and technology across borders, utilize energy resources in a cascade, build and share public facilities, and centrally and safely dispose of pollutants. High-end industrial agglomeration can enhance the efficiency of the vertical and horizontal division of labor between industries, form economies of scale and scope through sharing, complementarity, and learning effects, and effectively promote green high-end and “decarbonization” of urban industries.

Ecological restoration and reconstruction (*Ecolores*). This indicator is measured by the degree of urban green vegetation coverage [34]. The empirical test in Table 5 shows that the old industrial cities are vigorously promoting the transformation of urban areas and old industrial areas, making scientific use of the stock construction land and spatial layout, and encouraging the increased greenery. Old industrial cities also continue to accelerate the development and utilization of industrial heritage resources; actively carry out comprehensive management of the ecological environment in the form of afforestation and greening; promote the restoration of vegetation and soil, as well as water conservation, on abandoned industrial land. Old industrial cities are actively exploring the construction of creative blocks, new industrial parks, and green parks, guiding the development of green and low-carbon leading industries, and injecting strong impetus into green development.

### 6.2. Decoupling Degree Analysis

Drawing on the practice of Chen [35], the Tapio decoupling index model is used to test the decoupling elasticity coefficient of old industrial cities. The 94 old industrial cities are classified by year according to the above eight states.

**Figure 5:**
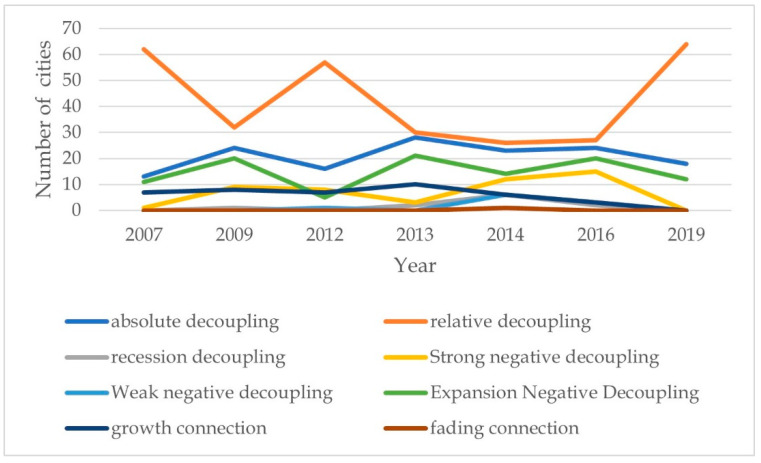
Number of cities decoupling states.

According to Figure 5, most of the old industrial cities in China are in a state of absolute decoupling and relative decoupling, showing a trend of intensive expansion and potential development. Following the implementation of the old industrial city adjustment and renovation policy in 2013, the number of cities that were absolutely decoupled increased by 12, which was significant compared with the previous year. It is worth noting that the number of recession decoupling cities shows an increasing trend and then abruptly decreases. It shows that after the implementation of the policy, the old industrial city has continuously strengthened the implementation of responsibility, supervision, and assessment; increased policy support; and strived to achieve the goal of adjustment and transformation.

According to their own development reality, old industrial cities should be responsible for managing the relationship between development and carbon emission reduction both in the short term and medium-to-long term. This requires formulating green development measures in a targeted manner, proposing a low-carbon development trajectory in line with urban conditions, and choosing reasonable goals. Old industrial cities must also pay attention to how they define emissions from the perspective of equity and efficiency, achieve high-quality urban economic development, and ensure that the goal of carbon peaking by 2030 can be achieved. In the future, the government should actively explore a multi-win path for low-carbon economic development, so as to achieve both high-quality economic growth and reduced carbon emission.

### 6.3. Environmental Hypothesis Validation

The Kuznets Environmental Hypothesis for Carbon Dioxide (CKC Hypothesis) refers to the fact that with the growth of the regional economy, there is a specific extreme point in the emission of carbon dioxide, which is an “inverted U-shaped” curve relationship that first increases and then decreases.

Taking carbon emissions as the dependent variable, the test results in Table 6 indicate a significant CO_2_ environmental Kuznets curve (EKC) in old industrial cities, and the “inverted U-shaped” hypothesis is clearly established. When the cubic term of per capita income is added to the continuous increase in per capita income in old industrial cities, carbon dioxide emissions still show a clear “inverted U-shaped” relationship, which initially increases slowly, and then continuously decreases; the “N-shaped” curve hypothesis does not hold. In reference to the general framework test of Lind [36], the maximum value of *Pgdp* is found to be significantly larger than the threshold, and the extreme value point tested is within the range of the sample data. The null hypothesis is rejected at the 10% significance level (P>|t|=0.0554), leaving an “inverted U-shape”.

According to the graphical analysis of the mean size, it can be seen that the sample of China’s old industrial cities has not yet crossed the inflection point. The increased intensity of environmental regulations and constraints has effectively curbed carbon emissions in the short term, and although the environmental quality has started to improve, there is still no significant reform effect of “reversely forcing carbon reduction” after crossing the inflection point. Incorporate carbon peaking and carbon neutrality-related indicators should also be factored into the comprehensive evaluation system for economic and social development. The government of the old industrial city should transform “lucid waters and lush mountains” into “invaluable mountains and silver mountains”, so as to achieve a win–win development pattern of both high economic quality and carbon and pollution reduction.

### 6.4. Evidence of Contamination Transfer

At the prefecture level, city governments implement, supervise, and regulate standards differently; the implementation of the adjustment and renovation policy of old industrial cities allows for the phenomenon of regional “pollution refuge”, such as “pollution passing on the neighborhood” and “carbon emissions harm others” [37]. Therefore, there is an increased likelihood that high-polluting, high-emission, and high energy-consuming enterprises will be located in the border region, resulting in the discharge of pollutants. This will not only affect the ecological environment of the moving-in place, but also cause “disaster” to its neighboring cities, causing serious problems of “two-for-nothing” or even “three-for-nothing” pollution in the junction area.

The test results in Table 6 show that the implementation of the policy will not cause an increase in the scale of carbon emissions in adjacent areas, nor at the expense of the quality of the ecological environment in adjacent cities. This is conducive to improving cooperation between cities across different regions, leading to common governance strategies within cities. The results obtained in this paper indirectly confirm the conclusions in Feng’s article [38]. Old industrial cities can improve their green total factor productivity without producing significant spatial spillover effects. Policy implementation can break the barrier that hinders regional cooperation and common governance. It is helpful for regions to strengthen and improve the institutionalization of benefit sharing, benefit compensation, and conflict mediation, which can be achieved through cooperation, elimination of administrative barriers and local protectionism.

In summary, the green and low-carbon effect of the old industrial city’s adjustment and renovation policy is generated from “self-innovation” rather than “pollution transfer”.

## 7. Conclusions and Policy Recommendations

### 7.1. Conclusions

The implementation of the old industrial city adjustment and renovation policy is a major project initiated by the Party Central Committee and the State Council to revitalize the green space and low-carbon transformation of old industrial cities. This paper uses data from 2006 to 2019 to obtain samples of 94 old industrial cities. Using the samples, this paper firstly analyzes the impact of the adjustment and renovation policies on the green space and carbon levels in old industrial cities, and then further analyzes the effective mechanism, decoupling model, and environmental hypothesis.

The following conclusions are drawn:

(1) Overall, the adjustment and renovation policies can produce a positive low-carbon and carbon-reduction effect, with an average reduction of about 0.068 units of carbon emissions. Compared with the average value, the implementation of the policy can reduce the urban carbon emissions by an average of about 310,670 tons. The policy implementation can effectively promote carbon reduction and pollution reduction in the middle quantile. There is no significant evidence that policy implementation leads to the transfer of pollution from old industrial cities to neighboring cities. The phenomenon of “blaming the neighbors” for serious pollution has not occurred, and thus the implementation of the policy can realize low-carbon regional development.
(2) The adjustment and renovation policies of old industrial cities have had a distinct and negative effect on pollution reduction and carbon reduction for sample cities in the eastern and western regions, large cities, and cities connected to high-speed rail.
(3) On the basis of summarizing excellent Chinese cases and conducting empirical estimates, it is found that after policy implementation, the innovation and improvement of urban green quality, the expansion of high-end industrial agglomeration scale, and the increase in ecological environment reconstruction are important mechanisms to reduce urban carbon emissions.
(4) There is a significant “inverted U-shaped” CO_2_ EKC in old industrial cities, but the “N-shaped” curve hypothesis does not hold. There are quite a few old industrial cities that have yet to cross the turning point of the EKC.
(5) During the implementation of the policy, in 2013, about 62% of the old industrial cities showed a state of relative decoupling and absolute decoupling. As the years pass, the trend of an increasing fluctuation of this ratio becomes more prominent, thus reversing the rapid growth of carbon dioxide in old industrial cities.

### 7.2. Policy Implications

Based on the empirical results and conclusion analysis, the following policy recommendations are put forward:

First, the city government should rationally design a dynamic path for carbon emission reduction to achieve absolute decoupling between economic growth and carbon emissions. City governments should fully integrate carbon peaking and carbon neutrality goals into medium- and long-term planning for economic and social development, and clarify the goals and outcomes of carbon reduction and carbon reduction tasks. City governments should cross the inflection point of the CKC curve as soon as possible; vigorously support cities with endowment conditions, key industries, and key enterprises to take the lead in peaking carbon emissions; and should not implement campaign-style carbon reduction. The government of the old industrial city should strengthen the top-level system design and promote the participation of diversified subjects, such as the government, society, and public, to realize the linkage of carbon emission reduction from top to bottom.

Second, the government should take various measures to achieve green and low-carbon development. The government should strengthen the leadership of cutting-edge innovation in the industrial base, cultivate compound green and low-carbon talents, implement key core technology innovations, cultivate green and low-carbon emerging industries, promote cross-sector and cross-industry collaborative innovation, and accelerate industrial low-carbon transformation and digital transformation. The government should further increase the proportion of renewable energy production and consumption, and provide an important guarantee for the construction of a zero-carbon green energy system. The government should cultivate advantageous high-end industrial clusters; improve the coverage of regional communication infrastructure; strengthen regional division of labor and efficient cooperation; broaden the rational flow of production factors and channels; strengthen the coupling and linkage of green and low-carbon industries; and promote the advanced industrial foundation of industrial cities, the specialization of industrial division of labor, and the modernization of industrial chains. The government should speed up the transformation of the original extensive and high-carbon development mode; strengthen the restoration and management of the geological environment and vegetation coverage; enhance the ability to conserve water sources and soil; and realize the coordination and unity of the economic, ecological, and social benefits of resource development.

Third, the government should build a cross-regional environmental coordination mechanism. The government should compile and apply a negative list for environmental access; implement an implementation plan for coordinating joint prevention, joint governance, and comprehensive governance; promote the simultaneous coordination of environmental regulations in adjacent areas; and strengthen the industrial division of labor and complementary advantages. The government should break the administrative level restrictions; build a cross-regional pollution-reduction and carbon-reduction coordination mechanism, and a cooperation platform; and achieve regional high-efficiency and integrated governance. The government should focus on the environmental effects of policy reforms in the central region cities, cities without high-speed rail, small cities, and medium-sized cities.

## Figures and Tables

**Table 1:** Descriptive statistics of the variables.

Variable	Mean	Sd	P50	Min	Max
*Lnco*2	5.7568	1.1184	5.6489	2.0727	9.6311
*Pergdp*	0.4029	0.4110	0.2805	0.0076	3.7777
*Oinvest*	0.9370	0.9177	0.7237	0.1197	16.9140
*Oeduc*	0.1717	0.2363	0.0872	0.0024	3.5022
*Upais*	2.2596	0.1436	2.2470	1.8312	2.8322
*Dmarket*	1.0861	0.6894	0.9195	0.0542	7.5041
*Teleinst*	0.8803	0.7019	0.7503	0.0161	9.7956

**Table 2:** Benchmark results estimation.

	(1) *Lnco*2	(2) *Lnco*2	(3) *Perco*2	(4) *Perco*2	(5) *Areco*2	(6) *Areco*2
*DID*	−0.068 ** (0.033)	−0.068 ** (0.033)	−0.017 ** (0.008)	−0.014 * (0.007)	−0.197 ** (0.091)	−0.190 ** (0.089)
*Pergdp*		0.155 *** (0.044)		0.084 *** (0.017)		0.588 (0.547)
*Oinvest*		−0.007 (0.005)		−0.001 (0.001)		0.029 (0.025)
*Oeduc*		−0.162 * (0.095)		−0.162 *** (0.042)		3.322 (2.972)
*Upais*		−0.139 (0.206)		0.071 (0.055)		−0.734 (0.793)
*Dmarket*		−0.004 (0.008)		−0.000 (0.002)		−0.028 (0.028)
*Teleinst*		−0.015 (0.021)		−0.047*** (0.008)		−0.302 (0.301)
_*Cons*	5.593 *** (0.006)	5.885 *** (0.459)	0.973 *** (0.002)	1.040 *** (0.124)	7.084 *** (0.017)	8.331 *** (1.616)
Year fixed effect	Yes	Yes	Yes	Yes	Yes	Yes
City fixed effect	Yes	Yes	Yes	Yes	Yes	Yes
City clustering	Yes	Yes	Yes	Yes	Yes	Yes
*N*	3570	3570	3570	3570	3570	3570
*R* ^2^	0.974	0.974	0.955	0.967	0.991	0.992

Note: Significant at the *** 1% level, ** 5% level, and * 10% level.

**Table 3:** Robustness test.

	Replacement Variable Test			Benchmark Variable Test		PSM-DID Test	
	*Lnco*2	*Lnco*2	*Lnco*2	*Lnco*2	*Lnco*2	*Lnco*2	*Lnco*2
*DID*	−0.070 ** (0.035)	−0.059 * (0.032)	−0.035 ** (0.014)	−0.067 ** (0.033)	−0.067 * (0.035)	−0.066 ** (0.033)	−0.066 * (0.034)
_*Cons*	5.759 *** (0.464)	5.652 *** (0.371)	2.630 *** (0.217)	5.885 *** (0.460)	5.885 *** (0.478)	5.899 *** (0.471)	5.898 *** (0.473)
Individual effect	Yes	Yes	Yes	Yes	Yes	Yes	Yes
Year effect	Yes	Yes	Yes	Yes	Yes	Yes	Yes
City clustering	Yes	Yes	Yes	Yes	Yes	Yes	Yes
Control variable	Yes	Yes	Yes	Yes	Yes	Yes	Yes
*N*	3315	3570	3060	3570	3570	3526	3515
*R* ^2^	0.970	0.969	0.988	0.969	0.967	0.968	0.968
**Instrumental variable test**			**Delete low-carbon city pilot samples**			**Group swap test**	
	***DID***	***Lnco*2**		***Lnco*2**	***Lnco*2**	***Lnco*2**	***Lnco*2**
Instrumental variable	0.103 *** (0.016)	−0.254 *** (0.059)	*DID*	−0.063 ** (0.032)	−0.061 * (0.031)	−0.017 (0.019)	0.007 (0.020)
_*Cons*	−0.498 *** (0.180)	4.022 *** (0.246)	_*Cons*	5.533 *** (0.007)	5.646 *** (0.334)	5.586 *** (0.006)	4.708 *** (0.532)
Control variable	Yes	Yes	Control variable	No	Yes	No	Yes
Year effect	Yes	Yes	Year effect	Yes	Yes	Yes	Yes
Individual effect	Yes	Yes	Individual effect	Yes	Yes	Yes	Yes
F value	18.29	-	City clustering	Yes	Yes	Yes	Yes
*N*	3570	3570	*N*	2184	2184	3570	3570
*R* ^2^	0.696	0.632	*R* ^2^	0.965	0.965	0.969	0.969

Note: *, **, and *** indicate significance at the 10%, 5%, and 1% level.

**Table 4:** Heterogeneity analysis.

	East Cities	Central Cities	West Cities	Large Cities	Medium Cities	Small Cities	High-Speed Rail Cities	No High-Speed Rail Cities
	(1) *Lnco*2	(2) *Lnco*2	(3) *Lnco*2	(4) *Lnco*2	(5) *Lnco*2	(6) *Lnco*2	(7) *Lnco*2	(8) *Lnco*2
*DID*	−0.079 ** (0.036)	−0.017 (0.043)	−0.123 * (0.073)	−0.156 *** (0.048)	−0.160 (0.110)	−0.059 ** (0.028)	−0.234 *** (0.045)	−0.147 ** (0.065)
_*Cons*	5.816 *** (0.442)	5.751 *** (0.523)	5.572 *** (1.195)	5.899 *** (1.112)	7.136 *** (0.831)	5.713 *** (0.293)	5.024 *** (0.512)	5.012 *** (0.769)
Individual effect	Yes	Yes	Yes	Yes	Yes	Yes	Yes	Yes
Year effect	Yes	Yes	Yes	Yes	Yes	Yes	Yes	Yes
City clustering	Yes	Yes	Yes	Yes	Yes	Yes	Yes	Yes
Control variable	Yes	Yes	Yes	Yes	Yes	Yes	Yes	Yes
*N*	1274	1260	1036	154	504	2912	2758	812
*R* ^2^	0.976	0.967	0.943	0.942	0.965	0.957	0.937	0.958

Note: *, **, and *** indicate significance at the 10%, 5%, and 1% level.

**Table 5:** Conduction path analysis.

	(1) *Grinnov*	(2) *Lnco*2	(3) *Hinsera*	(4) *Lnco*2	(5) *Ecolores*	(6) *Lnco*2
*DID*	0.174 *** (0.050)	−0.064 * (0.033)	0.069 *** (0.024)	−0.059 ** (0.007)	0.196 *** (0.059)	−0.039 ** (0.017)
*Grinnov*		−0.026 ** (0.013)				
*Hinsera*				−0.029 *** (0.010)		
*Ecolores*						−0.021 *** (0.007)
_*Cons*	4.471 *** (1.076)	5.985 *** (0.480)	10.281 *** (0.471)	6.187 *** (0.275)	5.573 *** (0.475)	5.907 *** (0.473)
Individual effect	Yes	Yes	Yes	Yes	Yes	Yes
Year effect	Yes	Yes	Yes	Yes	Yes	Yes
City clustering	Yes	Yes	Yes	Yes	Yes	Yes
Control variable	Yes	Yes	Yes	Yes	Yes	Yes
*N*	3570	3570	3570	3570	3570	3570
*R* ^2^	0.917	0.969	0.904	0.974	0.901	0.998

Note: *, **, and *** indicate significance at the 10%, 5%, and 1% level.

**Table 6:** Environmental hypothesis testing and evidence of contamination transfer.

	Environmental Hypothesis Testing			Pollution Transfer Test	
	(1) *Lnco*2	(2) *Lnco*2	(3) *Lnco*2	(4) *Lnco*2	(5) *Lnco*2
*DID*	−0.068 ** (0.033)	−0.068 ** (0.033)	−0.068 ** (0.033)	−0.046 (0.040)	−0.044 (0.029)
*Pgdp*	0.238 *** (0.075)	0.306 *** (0.101)	0.306 *** (0.101)		
*Pgdpsq*	−0.052 ** (0.022)	−0.061 ** (0.025)	−0.061 ** (0.025)		
*Pgdptr*			−0.014 (0.023)		
_*Cons*	5.515 *** (0.024)	5.872 *** (0.458)	5.904 *** (0.475)		
Individual effect	Yes	Yes	Yes	Yes	Yes
Year effect	Yes	Yes	Yes	Yes	Yes
City clustering	Yes	Yes	Yes	Yes	Yes
Control variable	No	Yes	Yes	No	Yes
*N*	3570	3570	3570	2254	2254
*R* ^2^	0.969	0.969	0.969	0.975	0.975

Note: *, **, and *** indicate significance at the 10%, 5%, and 1% level.

## Data Availability

Publicly available datasets were analyzed in this study. This data can data can be found here: https://pan.baidu.com/s/15_thRhUaUNevFgEMCs0jtg?pwd=1111 (accessed on 30 March 2022).
